# Early Community based Ayu-Emergency Intervention in Psychiatric Emergencies: A Community Based Participatory Research

**DOI:** 10.1192/j.eurpsy.2022.1505

**Published:** 2022-09-01

**Authors:** M. Voegeli, P. Sharma, S. Sharma, B. Sharma, I. Goyal, N. Sharma, S. Lakshmanan, A. Venu

**Affiliations:** 1 SAMU and CESU, Emergency Department, Mayotte, France; 2 AVP Research Foundation, Ayu-emergency, Coimbatore, India; 3 Samata Ayurved Prakoshtha, Ayurveda, Jaipur, India; 4 NMP Medical Research Institute, Integrative Therapies, Jaipur, India; 5 D’Ultimate Life Sciences, Ayurveda, Bhilwara, India; 6 Aarogyam (UK) CIC, Ayurveda Research, Leicester, United Kingdom

## Abstract

**Introduction:**

Psychiatry emergencies in India is major challenge for emergency service providers due to rapid growth of various behavioural, higher morbidity and mortality rate. Despite, psychiatry conditions are neglected area related to stigma, share, lack of awareness, and superstitious beliefs. There is an urgent need for specialist psychiatric emergency services, which can fill the huge gap between policymakers and health service providers joined together.

**Objectives:**

Present feasibility study has been undertaken to evaluate the safety and efficacy of combined emergency and Ayurveda medicine management of psychiatric emergencies in community-based settings.

**Methods:**

Ayu-Emergency Care project was developed in partnership with policy makers, researchers and health care providers, a collaborative platform of emergency medicine and Ayurveda medicine (Indian Traditional Medicine) for developing whole-system perspective, where providers work in a coordinated and joined-up way. Twenty trained care providers in psychiatry emergency and Ayurveda management worked in partnership with community-based organisation.

**Results:**

Patients with major clinical difficulties, in the acute phase were treated and managed by Ayu-Emergencypractitioners. Severe Agitation and violence relating to substance abuse, anxiety disorder and psychosis were the most common admission diagnoses. 2-weeks results indicate that Ayurveda intervention can reduce anxiety(p<0.01), aggression (p< 0.001) and agitation (p<0.01) significantly with no side effects reported. Intervention found to be clinically beneficial and cost-efficient alternative to out-of-home placements (i.e., Incarceration, psychiatric hospitalisation).

**Conclusions:**

The study’s findings highlight safety, efficacy and feasibility of intervention. Patients both prefer and seem to benefit from community-based ayu-psychiatric care, and early-intervention community program could be a good model for such care.

**Disclosure:**

No significant relationships.

